# Preterm intraventricular hemorrhage in vitro: modeling the cytopathology of the ventricular zone

**DOI:** 10.1186/s12987-020-00210-7

**Published:** 2020-07-20

**Authors:** Leandro Castaneyra-Ruiz, James P. McAllister, Diego M. Morales, Steven L. Brody, Albert M. Isaacs, David D. Limbrick

**Affiliations:** 1 Department of Neurological Surgery, Washington University School of Medicine and the St. Louis Children’s Hospital, Campus Box 8057, 660 South Euclid Ave., St. Louis, MO 63110 USA; 2 Department of Medicine, Washington University School of Medicine, St. Louis, MO 63110 USA; 3 Department of Neuroscience, Washington University School of Medicine, St. Louis, MO 63110 USA; 4 Department of Pediatrics, Washington University School of Medicine, St. Louis, MO 63110 USA

**Keywords:** Cell culture, Neural stem cells, Ependyma, Ventricular zone, Intraventricular hemorrhage, Post- hemorrhagic hydrocephalus, Preterm, Premature, Neonate

## Abstract

**Background:**

Severe intraventricular hemorrhage (IVH) is one of the most devastating neurological complications in preterm infants, with the majority suffering long-term neurological morbidity and up to 50% developing post-hemorrhagic hydrocephalus (PHH). Despite the importance of this disease, its cytopathological mechanisms are not well known. An in vitro model of IVH is required to investigate the effects of blood and its components on the developing ventricular zone (VZ) and its stem cell niche. To address this need, we developed a protocol from our accepted in vitro model to mimic the cytopathological conditions of IVH in the preterm infant.

**Methods:**

Maturing neuroepithelial cells from the VZ were harvested from the entire lateral ventricles of wild type C57BL/6 mice at 1–4 days of age and expanded in proliferation media for 3–5 days. At confluence, cells were re-plated onto 24-well plates in differentiation media to generate ependymal cells (EC). At approximately 3–5 days, which corresponded to the onset of EC differentiation based on the appearance of multiciliated cells, phosphate-buffered saline for controls or syngeneic whole blood for IVH was added to the EC surface. The cells were examined for the expression of EC markers of differentiation and maturation to qualitatively and quantitatively assess the effect of blood exposure on VZ transition from neuroepithelial cells to EC.

**Discussion:**

This protocol will allow investigators to test cytopathological mechanisms contributing to the pathology of IVH with high temporal resolution and query the impact of injury to the maturation of the VZ. This technique recapitulates features of normal maturation of the VZ in vitro, offering the capacity to investigate the developmental features of VZ biogenesis.

## Introduction

Intraventricular hemorrhage (IVH) is a severe neurological disorder of preterm infants, affecting about 20 percent of the preterm infants born at or below 32 gestational weeks [1]. Up to one half of infants with IVH develop post-hemorrhagic hydrocephalus (PHH), which is the most common etiology of pediatric hydrocephalus in North America [2]. Despite advances in the medical and surgical care of these infants, the prognosis of preterm IVH with PHH shows unacceptable high rates of persistent neurocognitive deficits (up to 85%) and cerebral palsy (up to 70%) [3]. Current treatment methods focus on draining cerebrospinal fluid (CSF) to reduce the excess pressure regardless of hydrocephalus etiology. There have been no substantive advances in the treatment of PHH in recent years, and few viable targeted therapeutic strategies have been proposed. To improve the care and outcomes for these patients, we must first define the fundamental mechanisms underlying the pathophysiology of IVH and its sequelae, and develop experimental models for testing therapeutic interventions.

An association between IVH, PHH, and neurodevelopmental impairment is well-established, but the mechanisms linking these disorders remain unclear. Recent evidence implicates impairment of cell junction complexes within the ventricular zone (VZ) [4–10] with associated ciliopathy in the etiology of congenital, non-hemorrhagic hydrocephalus [11–19] in both experimental models [4–6, 10, 11, 20–24] and humans [25–28]. VZ disruption is also identified as a key feature in IVH in humans [29]. Therefore, an understanding of the complex effects of the blood on the VZ is needed to develop preventive treatments for neurological sequelae of IVH/PHH. This protocol describe an in vitro experimental strategy that provides the ability to control the environment of murine VZ development. The reductionist approach allows a rapid and direct method to address questions related to mechanisms and effects of blood-related VZ disruption with high temporal and physiological resolution.

The methods detailed herein are uniquely applied to IVH, PHH, or hydrocephalus more generally. While other groups have cultured ependymal cells (EC) previously [30, 31], the notion of using VZ cultures to study the effects of IVH is a novel strategy reported from our group [32]. This protocol is a major step forward in IVH/PHH research, as it enables rigorously controlled mechanistic experiments, with the added flexibility of mimicking various human physiological parameters. For example, studies of the VZ from patients with PHH [29] and congenital hydrocephalus [27, 28] indicate that alterations in adherens junction molecules play an important role in EC damage. Furthermore, the VZ culture system eliminates problems such as drug bioavailability, the blood brain barrier, and it allows high-throughput testing of the effect of drugs. Testing of pharmacological agents in vitro is an important first step to not only assess efficacy but also determine optimal ranges of dosing prior in vivo testing.

It is well known that normal multiciliated EC differentiate from monociliated stem cells; however, the complex developmental mechanisms governing this process are still not completely clear. For example, how polarity is established in multiciliated EC and how trafficking of adhesion molecules to the cell membrane occurs, requires further study. Our model will be useful for the study of normal developmental neurobiological processes, the myriad of secondary newborn brain disorders (e.g. hypoxia, inflammation, nutrition and metabolic disorders), and therapeutics explorations designed to mitigate the neurological effects of VZ injury.

The VZ is a layer of tissue mainly composed of neuro-stem cells that line the fetal ventricular system [33, 34]. This layer is a proliferative area involved in the neurogenesis (intermediate progenitors, neurons) and gliogenesis (astrocytes, oligodendrocytes, ependymocytes) [35] of the brain; therefore representing an important platform for brain development. Our model enables investigation of the effect of blood on these precursor cells and specifically the impact on differentiation from VZ to multiciliated EC, the final step in the transition of the VZ. EC are involved in control of fluid movement between brain extracellular space and the CSF, in part to clear metabolites from the interstitial fluid [36]. EC also promote localized movement of fluid and its contents within the ventricles through the synchronized beating of cilia [37]. In fact, deficits in the formation of cilia cause serious disorders in the central nervous system, including hydrocephalus (reviewed in [38–40]). Despite the importance of the VZ in brain development, there are few reported methods to differentiate EC from brain neural stem cells (NSC) [30, 41–43]. We have recently reported on the effects of syngeneic blood on the VZ using an in vitro model [32], but the details of our procedures have not been published.

## Materials and methods

All procedures were approved by the Washington University Animal Care and Use Committee according to the Guide for the Care and Use of Laboratory Animals, NRC 2011.

### Telencephalic lateral wall dissection (modified [30] and [31])

#### Materials

- C57BL/6 mice age P0–P4 (Jackson Labs, Bar Harbor, ME).
- Mayo and Metzenbaum surgical scissors.
- Single-edge razor blade.
- Ultrafine surgical forceps (PT-31 super fine tip non-flex tweezers, Precise Hand Tools, London).
- Stereo microscope.
- Petri dish (10 cm^2^).
- Hank’s Balanced Salt Solution (HBSS) (#14170-112, Gibco, Waltham, MA), 1% penicillin, streptomycin, amphotericin B (PSA) (#30-004-CI, Corning Life Sciences, Corning, NY).

#### Procedure

**Fig. 1:**
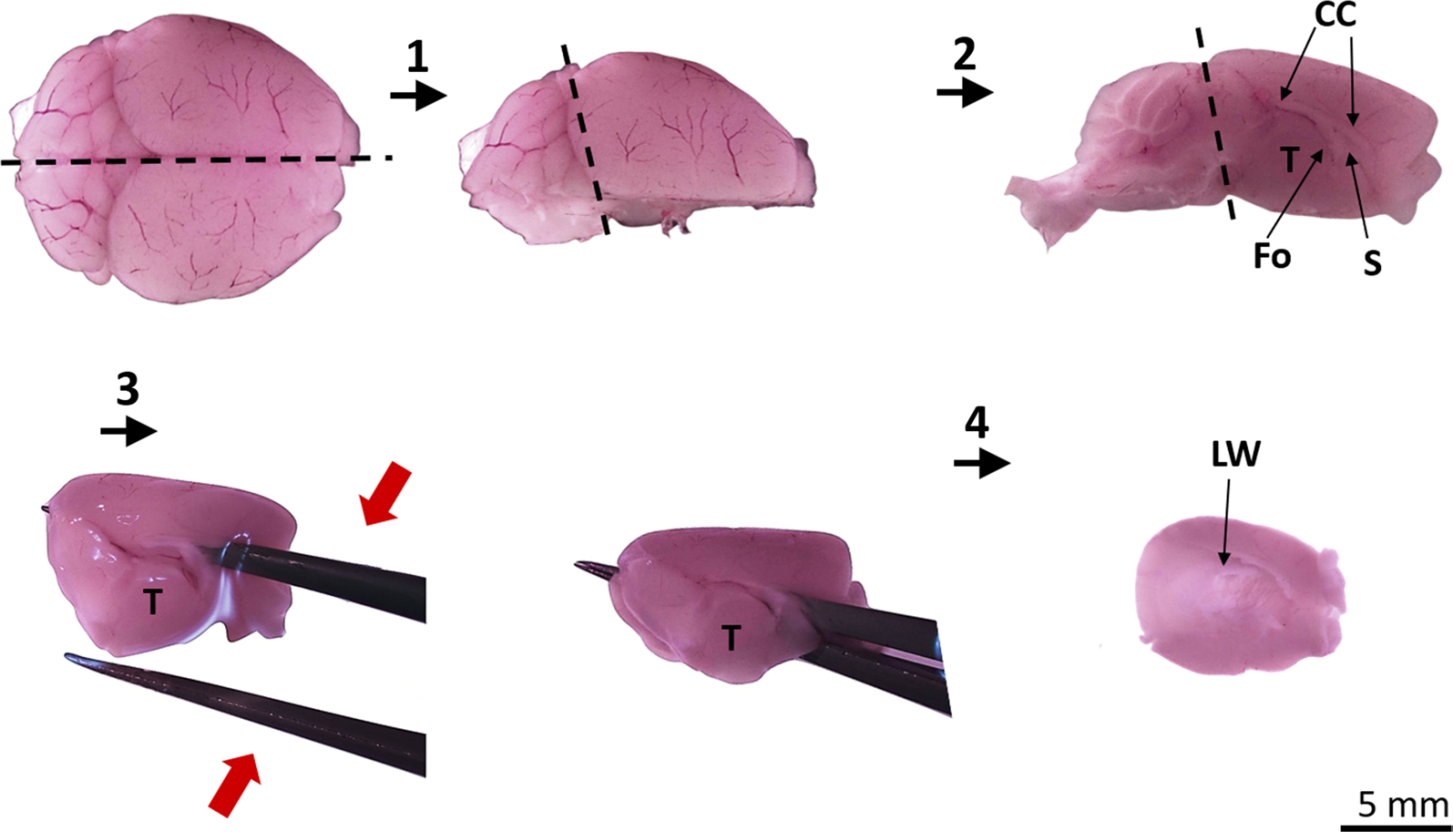
Dissection of the lateral ventricular wall to obtain VZ cells. Photographs of mouse brain gross morphology to illustrate isolation of the lateral ventricle wall using the following steps: (1) The brain is divided in the midline (dashed line) using a dorsal approach. (2) With the midsagittal surface down, the cerebral hemisphere is separated from the cerebellum (dashed line). (3) Beginning at the midsagittal surface, one sharp tip of ultrafine forceps is inserted into the dorsal lateral ventricle through the septum(s) between the anterior corpus callosum (CC) and the fornix (Fo), and angled so that the tip exits the occipital pole of the cerebral hemisphere; the other arm of the forceps should be external to the ventral surface of the brain. When the para-hippocampal gyrus is reached, the forceps are closed to detach the thalamus (T) and hippocampus medially from the lateral cerebral cortex. (4) By opening the lateral cerebral cortex, lateral wall of the ventricle (LW) is visualized and dissected to provide tissue for cell culture
- Position the medial hemispheric side superiorly and using ultrafine forceps, introduce one tip into the lateral ventricle in an anteroposterior direction all the way through the ventricle until the para-hippocampal gyrus is reached. Keep the opposite tip of the forceps in a ventral position regarding the anatomical orientation of the brain hemisphere, and close the forceps completely, producing an incision that divides the hippocampus and thalamus from the lateral wall. With a single, gentle twist of the wrist, remove the hippocampus and thalamus from the lateral wall (Fig. 1). Peel the meninges off the surface of the cerebral cortex, grasping them at the edge of the hemisphere and pulling away of the hemisphere. The wall dissection should not take longer than an hour and the tissue must not be stored or frozen before carrying out the VZ cell culture.

- Expose P0-P4 C57BL/6 mice to wet ice for 2 min or until the pup is not responsive or use methods as guided by the local IACUC for terminal studies. Once circulatory arrest by cooling is achieved, euthanize the pup by removing the head and gently strip the scalp.
- Remove the brain through a lateral incision from the foramen magnum toward the external auditory canal (~ 3 mm) and a superficial sagittal incision from the foramen magnum all the way through the midline using Metzenbaum surgical scissors. After that remove the frontal and parietal bones with curved surgical forceps.
- Deposit the brain in a Petri dish cooled in ice with HBSS, 1% PSA.
- Working under a stereomicroscope, dissect both cortical hemispheres, as well as the brainstem and the olfactory bulbs by cutting in the midline using a single-edge razor blade (Fig 1).

## Ventricular zone cell culture (modified from [30])

### Materials

- Poly-l-Lysine (P4707, Sigma, St. Louis, MO), Autoclaved H_2_O.
- Enzymatic solution: 1 mL Dulbecco’s Modified Eagle’s Medium (DMEM) 1% glutamax, 1% PSA, 17.1 µL papain (#37A17241, Worthington, Lakewood, OH), and 288 µg l-Cysteine (#C7352, Sigma, St. Louis, MO) for each brain dissected.
- Ca^2+^/Mg^2+^ free PBS Trypsin EDTA.
- Decomplemented FBS (heat inactivated, 55 °C one hour).
- Stop solution: 500 µL (10 mg/mL) of trypsin inhibitor (#10,109,878,001, Roche, Basel, Switzerland) in PBS (0.05% BSA) and 200 µL of DNAase I (#EN0521, Thermo Fisher Scientific, Waltham, MA) in 10 mL of L-15 medium (Leibovitz’s L15, #11415-064, Thermo Fisher Scientific, Waltham, MA) up to 9 brains.
- Proliferation medium: DMEM (#11965-092, Gibco, Waltham, MA), 1% glutamax (#35050-061, Gibco, Waltham, MA), 1% PSA (#MT 30-004-CI, Thermo Fisher Scientific, Waltham, MA), 10% Fetal Bovine Serum (FBS, #100-106, Gemini Bi-Products, Sacramento, CA).
- 15 mL Falcon tubes.
- Single-edge razor blades.
- Differentiation medium: DMEM, 1% glutamax, 1% PSA.
- Laminar flow hood.
- Tissue culture incubator 37 °C and 5% CO_2_.
- Centrifuge capable of holding 15 mL tubes.
- Hemocytometer.
- Inverted tissue culture microscope.
- 24-well tissue culture plate.
- Coverslips (#CLS1763012, 12 mm round coverslips, Chemglass Life Sciences, NJ).

### Procedure

- Coat a 25 cm^2^ flask with 1 mL Poly-l-Lysine for at least 1 h. Rinse the flask 3 times with autoclaved H_2_O and let dry under the laminar flow hood.
- Under a laminar flow hood, cut the dissected lateral walls in small pieces using the single edge razor blade until each piece can pass through a 1000 µL pipet tip.
- Deposit the tissue in a 15 mL Falcon tube and centrifuge for 1 min at 105×*g*. Remove the supernatant and add the enzymatic solution for 55 min at 37 °C and 5% CO_2_ within a standard tissue culture incubator.
- Centrifuge for 1 min 105×*g*, remove the supernatant and add the stop solution for 2 min.
- Centrifuge for 1 min 105×*g* and remove supernatant. Wash by adding 10 mL of L15.
- Centrifuge for 1 min 105×*g*, remove the supernatant and add 1 mL of L15 and disaggregate the cells by pipetting (no more 15 times).
- Centrifuge for 7 min 105×*g*, remove supernatant and add 1 mL of proliferation medium per brain used.
- Add three mL of proliferation medium with cells into every coated 25 cm^2^ flask and incubate at 37 °C and 5% CO_2_. Change the media the next day.
- When confluence reaches 80–100% (3–4 days), shake the flask at 250 RPM overnight and remove the medium to separate differentiated from non-differentiated cells (stem cells) remaining in the flask. Wash the cells in the flask three times with Ca^2+^/Mg^2+^ free PBS.
- Add 1 mL of trypsin EDTA for 5 min at 37 °C, 5% CO2 to detach the stem cells. After confirming that the cells have detached under the microscope, add 1 mL of decomplemented FBS to stop the trypsin activity. Avoid exposure to trypsin for more than 5 min.
- Quantify total number of cells using a hemocytometer. Centrifuge for 7 min 105×*g*, remove the supernatant and add proliferating media to a concentration of 2 × 10^6^ cells per mL.
- For further analysis by western blot, add 50 µL of the stem cells (10 × 10^4^ cells) into sterile 24 well plates; for analysis with immunocytochemistry, add a 25 µL (5 × 10^4^ cells) drop onto 12 mm coverslips inside the 24 well plates. (Pre-coated coverslips or wells with Poly-l-Lysine, see step [Coat a 25 cm^2^ flask with 1 mL Poly-l-Lysine…] in “Procedure” section). Incubate the drop for 1 h at 37 °C, 5% CO_2_; then add 1 mL of proliferative medium.
- On the next day, change the media to differentiation medium (1 mL). Renew the media every other day. From this point onward, the cells will progressively differentiate from stem cells to multiciliated EC. On days 3, 5, 7 and thereafter, the differentiation medium is added. About 4%, 23% and 52% of the cells, at days 3, 5, 7, respectively, should be multiciliated [32]. The cultures will remain stable for 10–12 days.
- After cervical dislocation, extract blood from 3-month-old mice through an orbital sinus puncture (reviewed by [44]; this age corresponds to the most feasible time to obtain the quantity of blood required for 24 well-plates. Other ages may be used depending on the design and clinical relevance of the experiment. To acquire syngeneic blood, the donors must be from the same C57BL/6 strain. The blood is held in a non-heparinized 50 mL Falcon tube on ice less than 5 min before placing 30 µL of whole blood or serum onto the media that is already in the well, over the VZ cell cultures. This step is carried out under the laminar flow cabinet in sterile conditions to mimic a bleeding process. Room temperature PBS 7.4 was used as control. To select a specific developmental time, consider that the cells are mostly NSC by 3 to 7 days, and mostly EC from 7 days-onward [32].

## In vitro model interpretation

While nearly any experimental method can be used in this model, to date we have primarily employed two analytical techniques:

### Western blot (WB)

- Rinse the cells three times with PBS. Add 60 µL RIPA (radioimmunoprecipitation assay buffer) solution (#R0278 Sigma, St.Louis, MO) onto the cells and carefully pipet multiple times (~ 10) to homogenize the cells. (Notice that WB requires a high concentration of proteins, therefore 10 × 10^4^ cells were plated for our VZ development; see step [For further analysis by western blot,…] in “Procedure” section. With this amount, it is possible to perform 3 mini gels WB per well of cells (loading ~ 20 µL of sample).
- Established WB routines can be followed to perform this technique; see Castaneyra et al. [32] and the list of antibodies used are available in Table 1.

**Table 1:** Immunohistochemical markers used to investigate ventricular zone and stem cells biology

Antibody	Host species	Dilution IHC	Dilution WB	Source
GFAP	Rabbit	1:300	1:1000	#7260, Abcam
βIV tubulin	Mouse	1:100	–	#11315, Abcam
AQP4	Rabbit	1:1000	–	#PAB 20767 Abnov
β-catenin	Mouse	1:100	–	#Ab16051 Abcam
S100β	Rabbit	1:100	–	#Ab52642, Abcam
N-cadherin	Mouse	1:25	1:50	#180224, Thermo Fisher Scientific
EMA	Mouse	1:100	–	#790-4463, Roche
GAPDH	Rabbit	–	1:500	#2118S, Cell Signaling Technology
Β-actin	Mouse	–	1:500	#A1978 Sigma
Antimouse Alexa fluor 555	Goat	1:300	–	#AQ21422, Thermo Fisher Scientific
Antirabbit Alexa Fluor 488	Goat	1:300	–	#A11034, Thermo Fisher Scientific
Peroxidase-conjugated antirabbit	–	–	1:10,000	#70,742, Cell Signaling Technology
Peroxidase-conjugated antimouse	–	–	1:5000	#SC-2005, Santa Cruz Biotechnology

Abcam, Cambridge, UK

Abnova, Taipei, Taiwan

Thermo Fisher Scientific, Waltham, MA

Cell Signaling Technology, Danvers, MA

Santa Cruz Biotechnology, Dallas, TX

### Immunohistochemistry (IHC)

- Rinse the cells three times with PBS, fix the cells cultured on coverslips with 4% paraformaldehyde in PBS PH 7.4 for 7 min. Wash three times with PBS to remove the remaining fixative. Note, that the fixative used should be optimized for the antibodies employed for IHC.
- Permeabilize the cells with 5% Bovine Serum Albumin in PBS, 1% Triton X-100 for 1 h.
- Established IHC routines can be followed to perform IHC, see [30, 32]. The list of antibodies used are available in Table 1.

## Image analysis

The immunofluorescent images were taken using Pascal confocal microscopy (Oberkochen, Germany). The roundness plug-in of ImageJ (NIH public software) was used to quantify the morphology of the cells labeled with anti-GFAP (see Additional file 1: Figure S1). The roundness is a magnitude that ranges from 1 when the shape is a perfect sphere to 0 when it is a line. For WB images, the molecular imager Chemidoc XRS and image lab 3.0 software were used to quantify band density (Bio-Rad, Hercules, CA).

## Statistical analysis

Student’s *t* test or Mann–Whitney *U*-test were used for parametric or non-parametric data respectively.

## Results

### EC differentiation

**Fig. 2:**
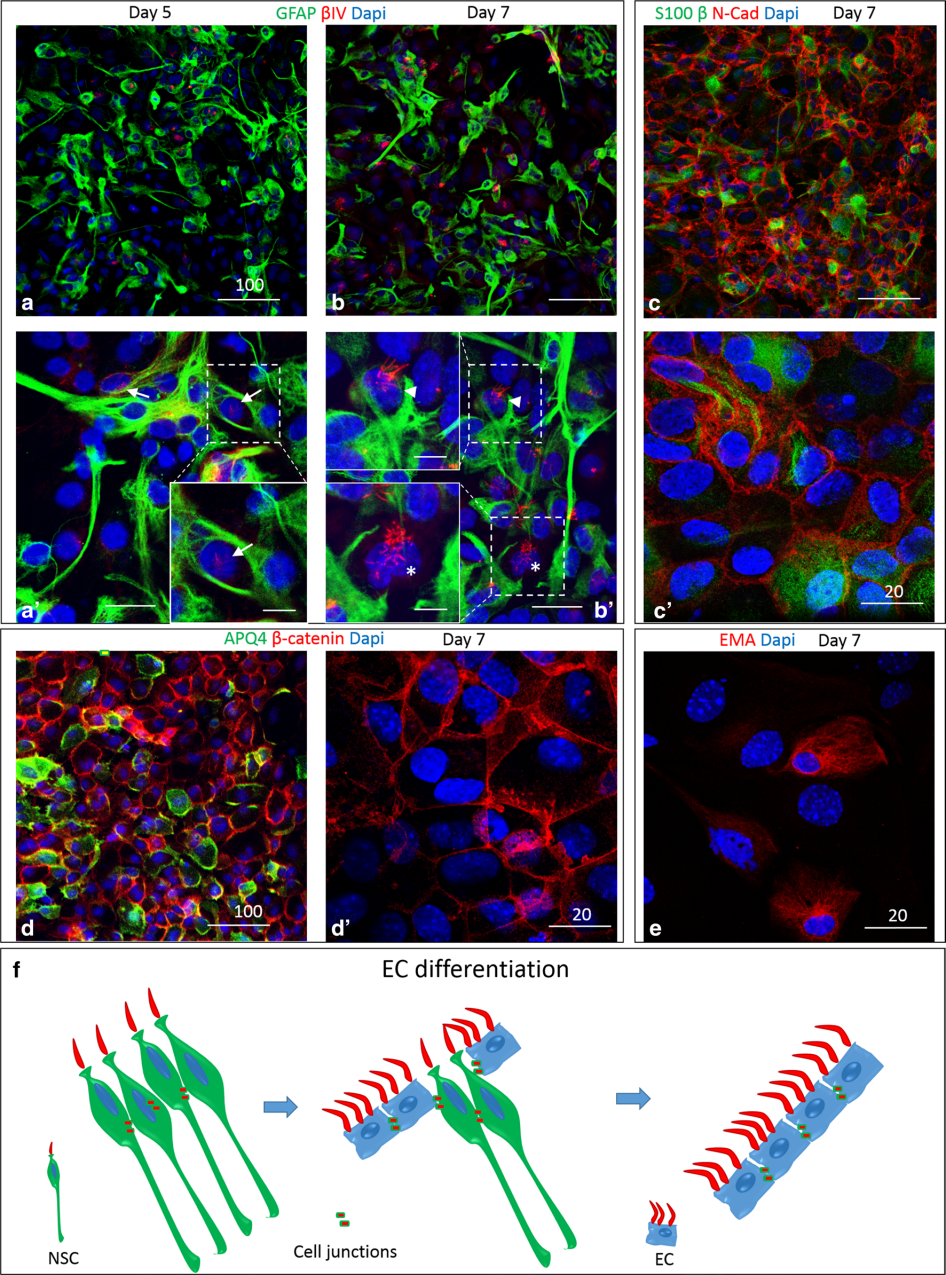
In vitro EC differentiation and characteristic markers: **a**, **b** Representative cell culture at day 5 and 7 respectively, labeled with anti-βIV (red) tubulin and anti-GFAP (green), in which a remarkable increase in βIV tubulin expression occurs at day 7. **a′**, **b′**. High magnification (×63) of the VZ at day 5 and 7 respectively. At day 7 the expression of βIV corresponds to multiciliated EC while at day 5 the labeling is linked to monociliated NSC. The inset in A’ shows a magnification of a monociliated NSC (arrow). The insets in B’ shows a multiciliated EC expressing GFAP (arrowhead) and a multiciliated EC not expressing GFAP (asterisk). **c**, **c′**. Representative VZ at day 7 immunolabeled with anti-S 100 β (green, associated with EC) and N-Cad (red, associated with both EC and NSC). **d**, **d′**. In vitro VZ at day 7 labeled with AQP4 (green) and β-Catenin (red). **e**. High magnification (×63) of the VZ labeled with EMA (mature EC marker). **f**. Schematic representation of the differentiation of the ependymal cells. Scale bars: **a**–**d** = 100 μm; **a′**, **b′ **= 25 μm; **c′**, **d′ **= 20 μm

VZ cells in vitro progressively differentiate from monociliated NSC to multiciliated EC [30, 32]. To define the development of the EC and monitor the validity this IVH in vitro model, we used well-known VZ/EC markers (Table 1). Since EC represent a glial lineage, characteristic astrocytic cell markers such as GFAP and S100β were present in the VZ (Fig. 2a–c′). S100β labels mature ependymal cells [45] and GFAP labels NSC and immature EC [9, 10, 29, 46]. Cilia markers are also appropriate to discriminate between NSC (monociliated) and EC (multiciliated) [47]. Double labeling with GFAP and βIV tubulin is appropriate in this model because allows distinction between NSC (if monociliated), immature EC (if multiciliated and positive for GFAP) and mature EC (if multiciliated and negative for GFAP) (Fig. 2a–b′). In this model the cells also express other markers such as epithelial membrane antigen (EMA) and Aquaporin 4 (AQP4) that show selective expression in mature ependymal cells (Fig. 2d, e). VZ/EC also elaborate cell junction proteins such as N-cadherin and β-catenin, indicating attachment and viability of the cell [48]; these proteins are expressed in both NSC and EC (Fig. 2c–d′). An increase in multiciliated ependymal cells over time is detected in control conditions (Fig. 2a–b′ and f). To confirm this, we quantified the percentage of cells expressing EMA on days 5 (37.51 ± 11.38), 6 (42.93 ± 10.77), and 7 (58.15 ± 10.66) (Fig. 4e, f); these percentages were similar to those previously reported by our group [32].

### Representative results of cell junction pathology

**Fig. 3:**
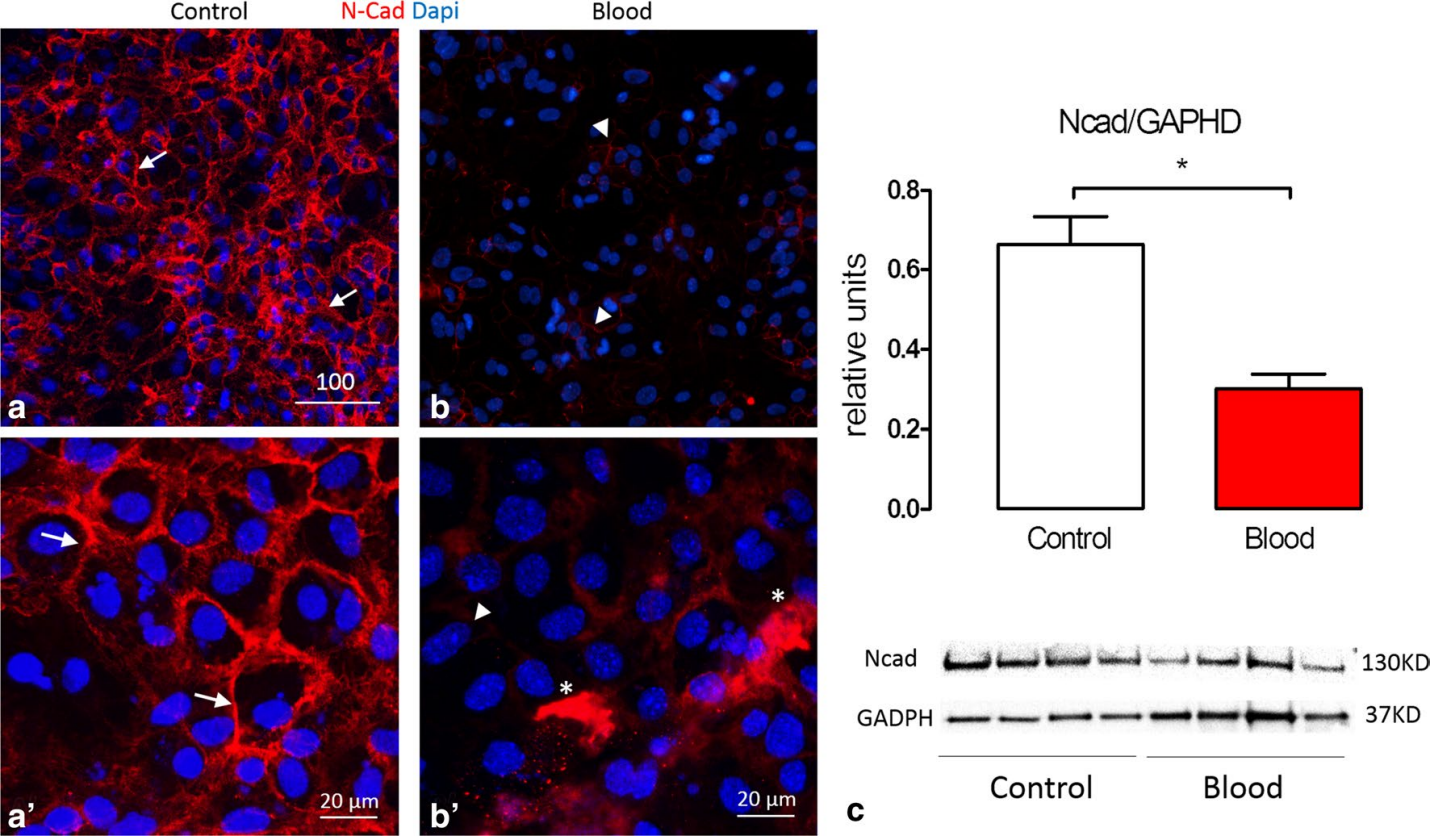
Cell junction impairments in the IVH in vitro model. **a**, **b** Comparisons of the expression of N-Cad at day 7 after 48 h of control (**a**) or blood (**b**). **a** Shows higher intensity of N-Cad expression and **b** a clear lack of N-Cad expression. **a′**, **b′** High magnification (×63) of **a**, **b** in which **a′** shows the typical lattice-like configuration of N-Cad vs **b′** that shows fragmentation (arrowhead), dislocation (asterisk) and diminution of the expression of N-Cad. **c** N-cad protein levels quantified by WB normalized with GADPH after 48 h of treatment; blood significantly decreases the expression of N-Cad (P < 0.05). Data are mean values from relative units (n = 8). Scale bars: **a**, **b** = 100 μm; **a′**, **b′ **= 20 μm

Impairment of cell junctions is a cardinal feature of IVH-related VZ disruption in human infants [29] and our in vitro model faithfully reproduces this phenomenon, with a decrease in the expression of N-cadherin after 48 h of blood exposure. Figure 3a–b′ illustrates the typical expression of adherens junctions in control (PBS) compared to blood-exposed cultures. There is diminution and dislocation of N-cadherin in blood-treated cultures, while control cultures show a characteristic polygonal shape as previously reported [42]. This model also allows the use of quantitative techniques such as WB to quantify targets of interest to understand this disease; Fig. 3c shows representative results of n-cadherin expression normalized with the expression of GADPH after 48 h in control and IVH conditions. In this case significant differences are found in the ratio of N-Cad/GADPH in control (0.66 ± 0.07) vs blood (0.30 ± 0.04) p < 0.05.

### Ventricular zone disruption and glial activation

**Fig. 4:**
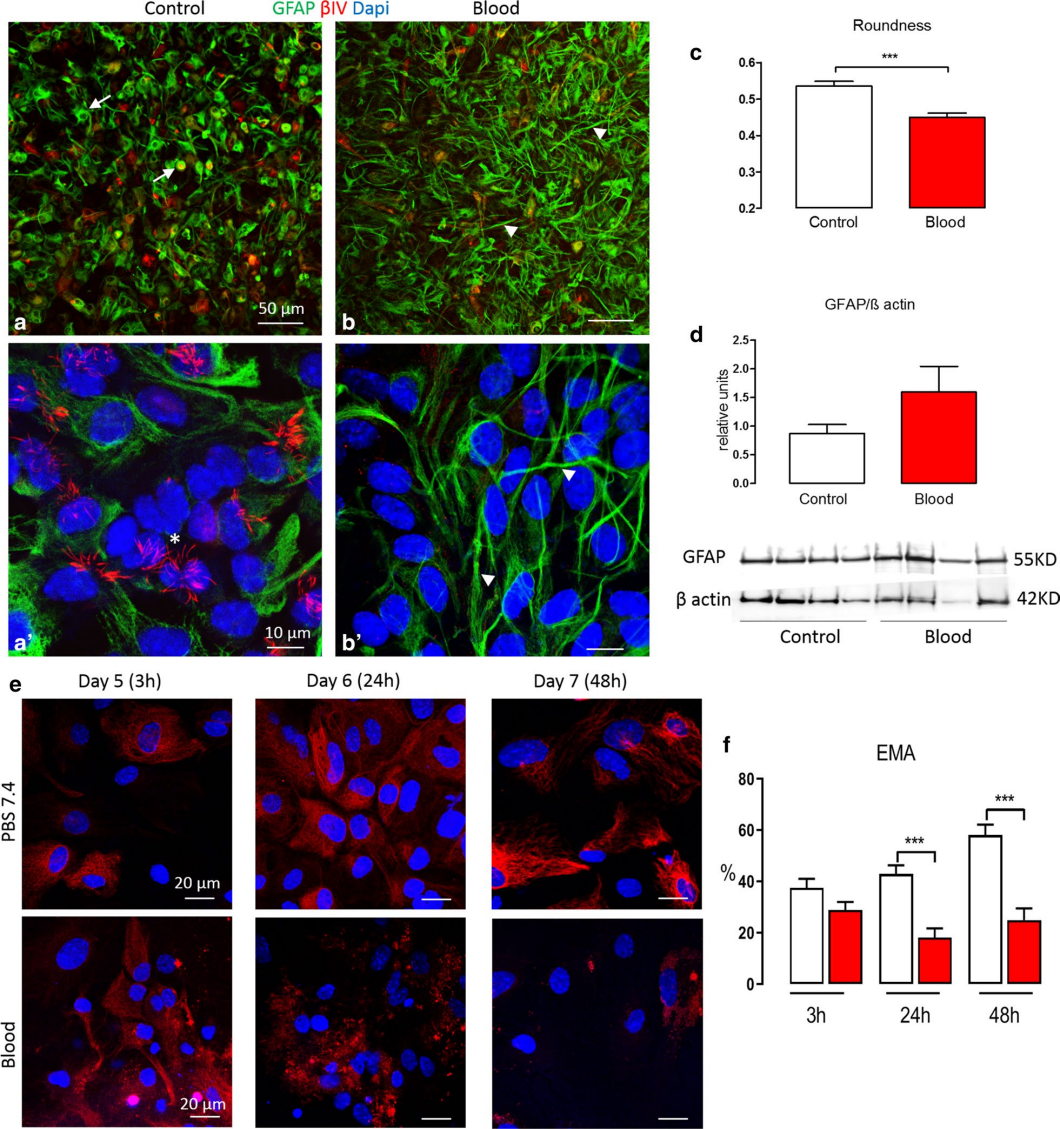
Glial activation and VZ disruption in the IVH in vitro model. **a**, **b** Comparisons of the expression of GFAP and βIV tubulin at day 7 after 48 h of control (**a**) or blood (**b**). **a** shows a qualitatively higher expression of βIV (arrows) and a lower expression of GFAP when compared to blood (**b**). **a′**, **b′** High magnification (×63) of **a**, **b** in which **a′** shows several multiciliated EC (asterisk) vs **b′** that shows a lack of multiciliated EC and changes in the morphology of the VZ cells with the increase of GFAP projections (arrowhead), as well as dislocation (asterisk) and diminution of the expression of N-Cad. **c** Graphical representation of the roundness of GFAP-labeled cells after 48 h of treatment (control vs. blood), In which the cells are significantly less round in blood conditions (p < 0.001). Data are mean of the roundness of 2 different experiments with at least n = 10 **d** GFAP protein levels quantified by WB normalized with β actin after 48 h of treatment, in which blood exposure increases the expression of GFAP. Data are mean from a single experiment with n = 8. **e** Comparisons of the expression of EMA at day 5 after 3 h of treatment at day 6 after 24 h of treatment and at day 7 after 48 h of treatment. The immunocytochemistry shows a decrease in the expression of EMA at 24 and 48 h after blood induction. **f** Quantification of EMA-positive cells. After 24 and 48 h treatment the mean percentage of cells expressing EMA was significantly decreased. Data are mean of the percentage of cells expressing EMA, from 2 experiments with at least n = 5 wells at 3, 24, and 48 hours, respectively (***p < 0.001; Student *t*-test). Scale bars: **a**, **b** = 50 μm; **a′**, **b′ **= 10 μm; **e** = 20 μm

Periventricular gliosis is another important feature of IVH/PHH in humans and in vivo models, and the VZ culture model also consistently demonstrates such glial activation [10, 29]. VZ disruption itself represents an alteration in the NSC/EC with a loss or detachment of these cells, followed by astroglial proliferation. Figure 4 shows a representative IHC in which a decrease in multiciliated EC, labeled with βIV tubulin, is present after 48 h of blood induction. The decrease of EC was confirmed by quantifying the expression of EMA after 3, 24 and 48 h in control VS the blood condition (3 h: 37.51 ± 11.38 VS 28.94 ± 11.14; 24 h: 42.93 ± 10.77 VS 18.18 ± 9.493; 48 h: 58.15 ± 10.66 VS 25.03 ± 16.31). Significant differences were observed at 24 and 48 h (p < 0.001) (Fig. 4e, f). There was also a change in the morphology of the cells expressing GFAP, with branch-like projections resembling reactive astrocytes (Fig. 4a–b′). To identify this astrocytosis, the morphology (roundness, measured using an ImageJ software application) of the cells seems to be a reliable variable in which the values close to 1 define a perfect sphere compared to values close to 0 which indicate a completely flat profile. Figure 3c shows a graphical representation of the roundness, being significantly lower after blood induction compared to control (respectively 0.44 ± 0.01 vs 0.53 ± 0.01 p < 0.001) due to the increase of elongated projections in blood conditions. The levels of GFAP can also be quantified through WB. Figure 3d shows results from a single WB of control compared to blood, in which an increase in the ratio of GFAP/β actin was found after 48 h of blood (control; 0.87 ± 0.16 vs blood; 1.60 ± 0.44), confirming that blood increases the amount of GFAP in the VZ. Other Inflammatory markers may be used to more completely understand these cytopathological mechanisms.

## Discussion

Despite the high prevalence and poor outcomes of PHH, the pathogenesis of this disorder remains poorly understood [49, 50]. One major factor limiting scientific progress in this field is a lack of appropriate models to probe the basic biology of the disease and to test novel therapeutic strategies. The current effort describes the protocol of the first in vitro model of preterm IVH/PHH and represents a major step forward for investigators. The in vitro IVH VZ model is a reliable tool to define the biological mechanisms involved in this devastating disorder.

To develop this model, an accurate isolation of the lateral wall of the lateral ventricle is a fundamental requirement. We have developed a straightforward and rapid technique to dissect the lateral ventricle by using two ultrafine forceps with just a single step to remove the thalamus, the medial wall of the lateral ventricle, and the hippocampus, versus the 5 steps previously described ([30, 31]).

The skull dissection must be done on cooled ice (Fig. 1) to provide enough stiffness to the fresh brain tissue; otherwise, the tissue may lose its shape and attach to the frontal and parietal bones complicating the bone removal.

To obtain a cell culture that mimics the cellular composition of the VZ with fidelity it is critical to totally remove the hippocampus and the meninges to prevent proliferation of neuronal-committed cells and fibroblasts, respectively. The cells are expanded for 3–5 days or until confluence. At this time point the differentiated cells are mechanically discarded. At the time the differentiation media is added, the cultures are composes of undifferentiated NSC that correspond to newborn lateral wall VZ development reported by Delgehyr et al. [30].

The most innovative process in this model is the addition of blood onto the cultured VZ to mimic the effects IVH on maturing neuroepithelial/ependymal cells. It is key to apply freshly obtained, whole blood onto the VZ cells as soon as possible (i.e., within 5 min). Within those 5 min, it is highly advised to keep the blood in ice cooled 25 mL falcon tubes to prevent coagulation. The presence of clots will impair pipetting the designated amount of blood onto the cells due to a pipet tip obstruction.

Likewise, this model is useful for studying the blood-related VZ cytopathology but does not take into account other physiological factors active in hydrocephalus such as increased intracranial pressure, which has been recently modeled using a 3-D neural cell cultures and a newly developed pressure controlled cell culture incubator [51].

While other authors have shown in vitro congenital hydrocephalus-dependent cytopathology [24, 42], this technique is a highly specific method to study the cytological mechanisms involved in the VZ after IVH. In this protocol paper, our aim is to describe our model [32] and show representative results. Despite only showing representative results from 3 to 48 h after blood induction, this technique provides a high temporal-resolution tool that opens the possibility to study the cellular response within seconds or minutes, which is problematic in vivo. This protocol also allows investigators to employ drugs to modulate and understand normal VZ development compared to IVH conditions without in vivo impediments, such us crossing the blood brain barrier in drug delivery or tissue dissections.

## Supplementary information

**Additional file 1: Figure S1.** ImageJ macroinstruction used to quantify the roundness of GFAP-positive cells. **A**. Code of the macroinstruction that allows the quantification of the roundness of the cells in a single step. **B.** Representative Image of the cell cultures labeled with GFAP after running the macroinstruction. Note that the macroinstruction does not count cells touching the edges of the image.

## Data Availability

Not applicable.

## References

[1] Stoll BJ, Hansen NI, Bell EF, Walsh MC, Carlo WA, Shankaran S (2015). Trends in care practices, morbidity, and mortality of extremely preterm neonates, 1993–2012. JAMA.

[2] Christian EA, Jin DL, Attenello F, Wen T, Cen S, Mack WJ (2016). Trends in hospitalization of preterm infants with intraventricular hemorrhage and hydrocephalus in the United States, 2000–2010. J Neurosurg Pediatr..

[3] Adams-Chapman I, Hansen NI, Stoll BJ, Higgins R, Network NR (2008). Neurodevelopmental outcome of extremely low birth weight infants with posthemorrhagic hydrocephalus requiring shunt insertion. Pediatrics.

[4] Jimenez AJ, Garcia-Verdugo JM, Gonzalez CA, Batiz LF, Rodriguez-Perez LM, Paez P (2009). Disruption of the neurogenic niche in the subventricular zone of postnatal hydrocephalic hyh mice. J Neuropathol Exp Neurol.

[5] Paez P, Batiz LF, Roales-Bujan R, Rodriguez-Perez LM, Rodriguez S, Jimenez AJ (2007). Patterned neuropathologic events occurring in hyh congenital hydrocephalic mutant mice. J Neuropathol Exp Neurol.

[6] Batiz LF, Paez P, Jimenez AJ, Rodriguez S, Wagner C, Perez-Figares JM (2006). Heterogeneous expression of hydrocephalic phenotype in the hyh mice carrying a point mutation in alpha-SNAP. Neurobiol Dis.

[7] Perez-Figares JM, Jimenez AJ, Perez-Martin M, Fernandez-Llebrez P, Cifuentes M, Riera P (1998). Spontaneous congenital hydrocephalus in the mutant mouse hyh. Changes in the ventricular system and the subcommissural organ. J Neuropathol Exp Neurol.

[8] Wagner C, Batiz LF, Rodriguez S, Jimenez AJ, Paez P, Tome M (2003). Cellular mechanisms involved in the stenosis and obliteration of the cerebral aqueduct of hyh mutant mice developing congenital hydrocephalus. J Neuropathol Exp Neurol.

[9] Batiz LF, Jimenez AJ, Guerra M, Rodriguez-Perez LM, Toledo CD, Vio K (2011). New ependymal cells are born postnatally in two discrete regions of the mouse brain and support ventricular enlargement in hydrocephalus. Acta Neuropathol.

[10] Roales-Bujan R, Paez P, Guerra M, Rodriguez S, Vio K, Ho-Plagaro A (2012). Astrocytes acquire morphological and functional characteristics of ependymal cells following disruption of ependyma in hydrocephalus. Acta Neuropathol.

[11] Guerra MM, Henzi R, Ortloff A, Lichtin N, Vio K, Jimenez AJ (2015). Cell junction pathology of neural stem cells is associated with ventricular zone disruption, hydrocephalus, and abnormal neurogenesis. J Neuropathol Exp Neurol.

[12] Milhorat TH, Hammock MK, Breckbill DL (1975). Acute unilateral hydrocephalus resulting from oedematous occlusion of foramen of Monro: complication of intraventricular surgery. J Neurol Neurosurg Psychiatry.

[13] Fame RM, Chang JT, Hong A, Aponte-Santiago NA, Sive H (2016). Directional cerebrospinal fluid movement between brain ventricles in larval zebrafish. Fluids Barriers CNS..

[14] Cifuentes M, Rodriguez S, Perez J, Grondona JM, Rodriguez EM, Fernandez-Llebrez P (1994). Decreased cerebrospinal fluid flow through the central canal of the spinal cord of rats immunologically deprived of Reissner’s fibre. Exp Brain Res.

[15] Swiderski RE, Agassandian K, Ross JL, Bugge K, Cassell MD, Yeaman C (2012). Structural defects in cilia of the choroid plexus, subfornical organ and ventricular ependyma are associated with ventriculomegaly. Fluids Barriers CNS..

[16] Appelbe OK, Bollman B, Attarwala A, Triebes LA, Muniz-Talavera H, Curry DJ (2013). Disruption of the mouse Jhy gene causes abnormal ciliary microtubule patterning and juvenile hydrocephalus. Dev Biol..

[17] Worthington WC, Cathcart RS (1966). Ciliary currents on ependymal surfaces. Ann N Y Acad Sci.

[18] Greenstone M, Cole PJ (1985). Ciliary function in health and disease. Br J Dis Chest..

[19] Afzelius BA (2004). Cilia-related diseases. J Pathol.

[20] Ortloff AR, Vio K, Guerra M, Jaramillo K, Kaehne T, Jones H (2013). Role of the subcommissural organ in the pathogenesis of congenital hydrocephalus in the HTx rat. Cell Tissue Res.

[21] Rodriguez EM, Guerra MM, Vio K, Gonzalez C, Ortloff A, Batiz LF (2012). A cell junction pathology of neural stem cells leads to abnormal neurogenesis and hydrocephalus. Biol Res.

[22] Ferland RJ, Batiz LF, Neal J, Lian G, Bundock E, Lu J (2009). Disruption of neural progenitors along the ventricular and subventricular zones in periventricular heterotopia. Hum Mol Genet.

[23] Dominguez-Pinos MD, Paez P, Jimenez AJ, Weil B, Arraez MA, Perez-Figares JM (2005). Ependymal denudation and alterations of the subventricular zone occur in human fetuses with a moderate communicating hydrocephalus. J Neuropathol Exp Neurol.

[24] Henzi R, Vío K, Jara C, Johanson CE, McAllister JP, Rodríguez EM (2020). Neural stem cell therapy of foetal onset hydrocephalus using the HTx rat as experimental model. Cell Tissue Res.

[25] Acabchuk RL, Sun Y, Wolferz R, Eastman MB, Lennington JB, Shook BA (2015). 3D modeling of the lateral ventricles and histological characterization of periventricular tissue in humans and mouse. J Vis Exp..

[26] Shook BA, Lennington JB, Acabchuk RL, Halling M, Sun Y, Peters J (2014). Ventriculomegaly associated with ependymal gliosis and declines in barrier integrity in the aging human and mouse brain. Aging Cell.

[27] Sival DA, Guerra M, den Dunnen WF, Batiz LF, Alvial G, Castaneyra-Perdomo A (2011). Neuroependymal denudation is in progress in full-term human foetal spina bifida aperta. Brain Pathol.

[28] Ortega E, Munoz RI, Luza N, Guerra F, Guerra M, Vio K (2016). The value of early and comprehensive diagnoses in a human fetus with hydrocephalus and progressive obliteration of the aqueduct of Sylvius: case Report. BMC Neurol..

[29] McAllister JP, Guerra MM, Ruiz LC, Jimenez AJ, Dominguez-Pinos D, Sival D (2017). Ventricular zone disruption in human neonates with intraventricular hemorrhage. J Neuropathol Exp Neurol.

[30] Delgehyr N, Meunier A, Faucourt M, Bosch Grau M, Strehl L, Janke C (2015). Ependymal cell differentiation, from monociliated to multiciliated cells. Methods Cell Biol.

[31] Grondona JM, Granados-Duran P, Fernandez-Llebrez P, Lopez-Avalos MD (2013). A simple method to obtain pure cultures of multiciliated ependymal cells from adult rodents. Histochem Cell Biol.

[32] Castaneyra-Ruiz L, Morales DM, McAllister JP, Brody SL, Isaacs AM, Strahle JM (2018). Blood exposure causes ventricular zone disruption and glial activation in vitro. J Neuropathol Exp Neurol.

[33] Rakic P (2009). Evolution of the neocortex: a perspective from developmental biology. Nat Rev Neurosci.

[34] Noctor SC, Flint AC, Weissman TA, Dammerman RS, Kriegstein AR (2001). Neurons derived from radial glial cells establish radial units in neocortex. Nature.

[35] Rowitch DH, Kriegstein AR (2010). Developmental genetics of vertebrate glial-cell specification. Nature.

[36] Del Bigio MR (2010). Ependymal cells: biology and pathology. Acta Neuropathol.

[37] Faubel R, Westendorf C, Bodenschatz E, Eichele G (2016). Cilia-based flow network in the brain ventricles. Science.

[38] Jimenez AJ, Dominguez-Pinos MD, Guerra MM, Fernandez-Llebrez P, Perez-Figares JM (2014). Structure and function of the ependymal barrier and diseases associated with ependyma disruption. Tissue Barriers..

[39] Zappaterra MW, Lehtinen MK (2012). The cerebrospinal fluid: regulator of neurogenesis, behavior, and beyond. Cell Mol Life Sci..

[40] Sharma N, Berbari NF, Yoder BK (2008). Ciliary dysfunction in developmental abnormalities and diseases. Curr Top Dev Biol.

[41] Weibel M, Pettmann B, Artault JC, Sensenbrenner M, Labourdette G (1986). Primary culture of rat ependymal cells in serum-free defined medium. Brain Res.

[42] Prothmann C, Wellard J, Berger J, Hamprecht B, Verleysdonk S (2001). Primary cultures as a model for studying ependymal functions: glycogen metabolism in ependymal cells. Brain Res.

[43] Henzi R, Guerra M, Vio K, Gonzalez C, Herrera C, McAllister P (2018). Neurospheres from neural stem/neural progenitor cells (NSPCs) of non-hydrocephalic HTx rats produce neurons, astrocytes and multiciliated ependyma: the cerebrospinal fluid of normal and hydrocephalic rats supports such a differentiation. Cell Tissue Res.

[44] Parasuraman S, Raveendran R, Kesavan R (2010). Blood sample collection in small laboratory animals. J Pharmacol Pharmacother..

[45] Steiner J, Bernstein HG, Bielau H, Berndt A, Brisch R, Mawrin C (2007). Evidence for a wide extra-astrocytic distribution of S100B in human brain. BMC Neurosci..

[46] Rodriguez EM, Guerra MM (2017). Neural stem cells and fetal-onset hydrocephalus. Pediatr Neurosurg.

[47] Kriegstein A, Alvarez-Buylla A (2009). The glial nature of embryonic and adult neural stem cells. Annu Rev Neurosci.

[48] Miyamoto Y, Sakane F, Hashimoto K (2015). N-cadherin-based adherens junction regulates the maintenance, proliferation, and differentiation of neural progenitor cells during development. Cell Adhes Migrat..

[49] Garton T, Keep RF, Wilkinson DA, Strahle JM, Hua Y, Garton HJ (2016). Intraventricular hemorrhage: the role of blood components in secondary injury and hydrocephalus. Transl Stroke Res..

[50] Strahle J, Garton HJ, Maher CO, Muraszko KM, Keep RF, Xi G (2012). Mechanisms of hydrocephalus after neonatal and adult intraventricular hemorrhage. Transl Stroke Res..

[51] Smith ME, Eskandari R (2018). A novel technology to model pressure-induced cellular injuries in the brain. J Neurosci Methods.

