# Supplementary figures and images for: Preterm intraventricular hemorrhage in vitro: modeling the cytopathology of the ventricular zone
Source: Fluids Barriers CNS. 2020 Jul 20;17:46. doi: 10.1186/s12987-020-00210-7 (PMC7372876; doi:10.1186/s12987-020-00210-7)

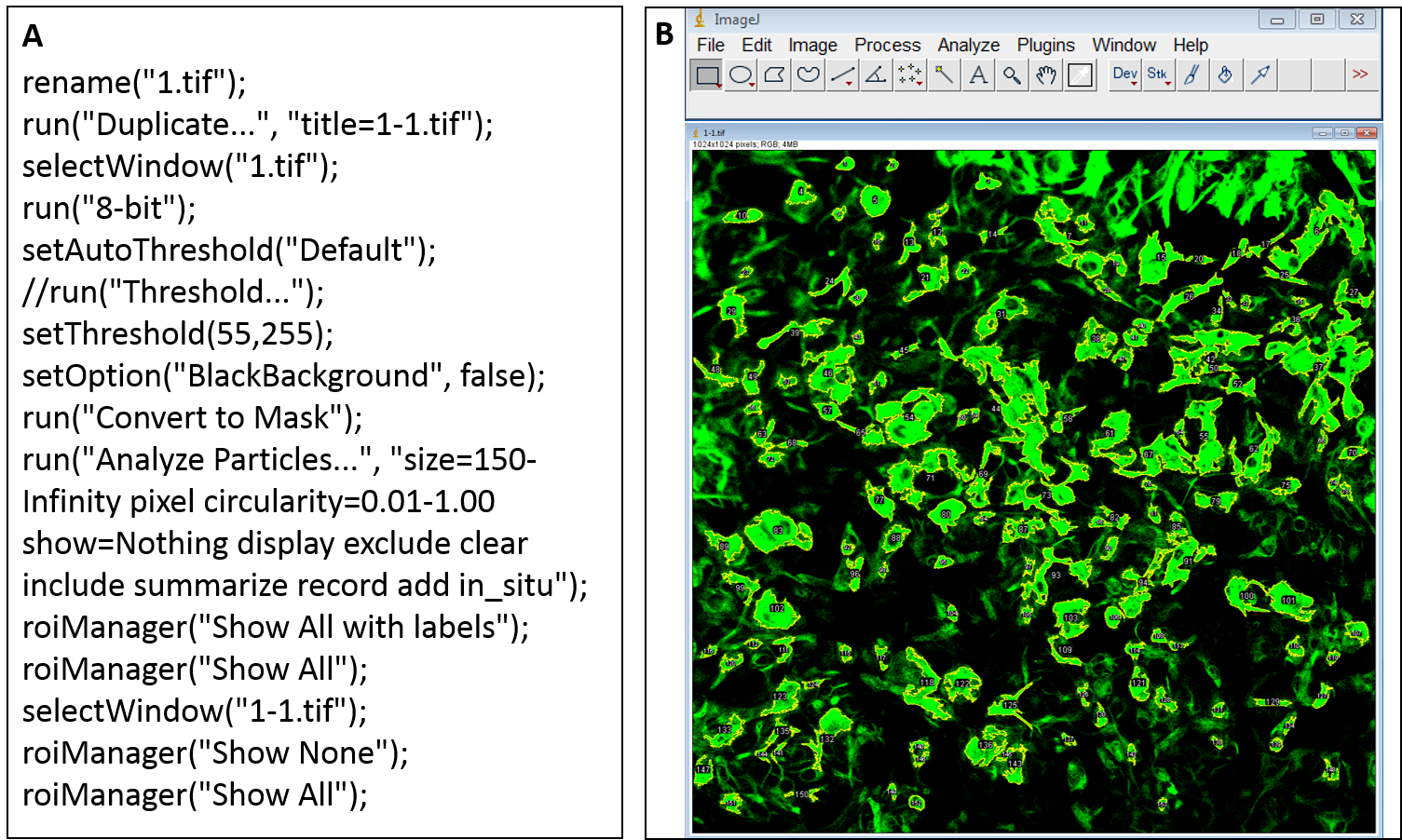

Supplement: Supplementary file 1 — Additional file 1: Figure S1. ImageJ macroinstruction used to quantify the roundness of GFAP-positive cells. A. Code of the macroinstruction that allows the quantification of the roundness of the cells in a single step. B. Representative Image of the cell cultures labeled with GFAP after running the macroinstruction. Note that the macroinstruction does not count cells touching the edges of the image. [file 12987_2020_210_MOESM1_ESM.tif]
